# Supplementary material for: MCell4 with BioNetGen: A Monte Carlo simulator of rule-based reaction-diffusion systems with Python interface
Source: PLoS Comput Biol. 2024 Apr 24;20(4):e1011800. doi: 10.1371/journal.pcbi.1011800 (PMC11073787; doi:10.1371/journal.pcbi.1011800)
Supplement: S1 Text — (PDF) [file pcbi.1011800.s001.pdf]

Supporting Information Text S1 for MCell4 with BioNetGen: A Monte Carlo Simulator of Rule-Based Reaction-Diffusion Systems with Python Interface

Adam Husar[1], Mariam Ordyan[2], Guadalupe C. Garcia[1], Joel G. Yancey[1], Ali S. Saglam[3], James R. Faeder[3], Thomas M. Bartol[1,*], Mary B. Kennedy[4], Terrence J. Sejnowski[1,2]

April 3, 2024

1 Computational Neurobiology Lab, Salk Institute for Biological Studies, California, La Jolla 92037, United States of America
2 Institute for Neural Computations, University of California, San Diego, California, La Jolla, 92037, United States of America
3 Department of Computational and Systems Biology, University of Pittsburgh, Pittsburgh, PA 15260, United States of America
4 Division of Biology and Biological Engineering, California Institute of Technology, Pasadena, California 91125, United States of America

* Corresponding author: bartol@salk.edu

5 Supporting Information

5.1 MCell3/MCell3-R and MCell4 Features Comparison

MCell4 was designed with the aim of backward compatibility with every existing MCell3 and MCell3-R model, but there are some notable differences described in Table A.

Feature	MCell3/MCell3-R	MCell4
Model programmability	Static definition in MDL. Users can dynamically change certain aspects of model by via checkpointing simulation state and reloading state with changed parameters	Arbitrary model modifications at run-time between each iteration and event-driven callbacks can act immediately within iteration time (e.g., Fig 21)
Reactions and reactive surface hits	MDL syntax allows the release of molecules after a reaction or when a molecule hits a surface	Python callback functions can be registered to occur at any reaction event or molecule/surface hit event and cause the release of molecules (e.g., Section 3.1.2), firing of other reactions, calling of external physics engines, etc.
Periodic boundary conditions (PBC)	Traditional PBC where molecules wrap around when they hit an edge of boundary, and mirrored PBC where the geometry is mirrored in adjacent boxes	Not supported directly but can be modeled by reactive surface hits and callbacks where Python code defines where to move the molecule
Checkpointing	Checkpoints at specified iteration and at real time	Checkpoints at specified iteration and asynchronous checkpoint triggered by Python code
BNGL species	Unlimited number of species, no cleanup of unused species	Unlimited number of species, periodic cleanup of unused species (section 2.4)
BNGL reactions	Using Nfsim to maintain reaction sub-network, no cleanup of generated reactions	Using libBNG to maintain reaction sub-network, periodic cleanup of generated reactions
BNGL model export	Partial, reaction rules must be in CellBlender not in MDL	Partial, exported from a Python model (section 2.4.2)
Reaction rate units	Only MCell units	MCell and BioNetGen units (Table 1)
Dynamic geometry	Predefined changes of geometry through CellBlender, scripting, or manual entry	Python code can introspect positions of vertices and set new vertex positions for a new iteration, surface can be set as immovable, partial detection of surface to surface collisions that block moving of one of the surfaces (supported when only one of the surfaces move) [1]
Surface-surface molecule reactions	Only between molecules on the same surface	Basic support for reactions between molecules on two nearby surfaces with configurable reaction radius
Warnings, Notifications and Reports	Detailed, many options (see section 3.1 of [2])	Limited compared to MCell3-R: High reaction probability, varying reaction probability, surface overlaps, reactions and species report

Table A: List of main differences in features between MCell3/MCell3-R and MCell4.

5.2 Supported BioNetGen Features and Keywords in libBNG

Library libBNG is a new C++17 library developed as a part of MCell4. It offers API that allows for it to be used outside fo MCell4. It features a Flex/Bison (e.g., [3]) parser that transforms the input BioNetGen language (BNGL) representation into an abstract syntax tree (AST), performs semantic checks, and then converts this AST into an internal C++ representation. As the main goal was to support MCell4, not all features of the BioNetGen suite are supported. Table B lists the keywords of BNGL supported in libBNG.

BNGL keyword	Meaning, use in MCell4
parameters	Denotes section with constant parameters. Symbols with special prefix MCELL_DIFFUSION_CONSTANT_2D_ and MCELL_DIFFUSION_CONSTANT_3D_ define whether an (elementary) molecule is a surface (2D) or volume (3D) molecule and this symbol's value is the molecule's diffusion constant. Another special prefix MCELL_REDEFINE_ allows redefining a symbol's value when it is interpreted by MCell4.
molecule types	Denotes a section with elementary molecule types.
species	Initial molecule releases, allows specification of the compartment and number of molecules to release.
compartments	Defines volumetric and surface compartments, with their volume and parent/child relationship hierarchy, modeled as cubes and associated surfaces in MCell4.
observables	Defines which species patterns will be counted at run-time, statement "Molecules" counts each occurrence a molecule within a matching pattern, "Species" counts a complex matching the pattern a maximum of one time.
reaction rules	Defines reaction rules, unidirectional or reversible, only constants or constant parameters can be used as reaction rates.
actions	Used to specify which simulator to run in BioNetGen, ignored in MCell4.

Table B: Supported features of BNGL in libBGL and their intepretation in MCell4.

References

1. MCell4 DynamicGeometry Documentation;. `https://mcell.org/mcell4_documentation/features/dynamic_geometry.html`.
2. MCell Quick Reference Guide;. `https://mcell.org/documentation/qrg/index.html#mcell-quick-reference-guide`.
3. Levine J, John L. Flex & Bison. 1st ed. O'Reilly Media, Inc.; 2009.
