# Supplementary material for: MCell4 with BioNetGen: A Monte Carlo simulator of rule-based reaction-diffusion systems with Python interface
Source: PLoS Comput Biol. 2024 Apr 24;20(4):e1011800. doi: 10.1371/journal.pcbi.1011800 (PMC11073787; doi:10.1371/journal.pcbi.1011800)
Supplement: Attachment — Submitted filename: review_PLOS_CB_final.pdf [file pcbi.1011800.s002.pdf]

Review of Adam Husar, Mariam Ordyan, Guadalupe C. Garcia, Joel G. Yancey, Ali S. Saglam, James R. Faeder, Thomas M. Bartol, Terrence J. Sejnowski, *"MCELL4 WITH BIONETGEN: A MONTE CARLO SIMULATOR OF RULE-BASED REACTION-DIFFUSION SYSTEMS WITH PYTHON INTERFACE"*

The authors report a major new version of the stochastic simulation software MCell. MCell supports particle-based spatial models of biochemical systems. The new version, MCell4, features a Python API that opens the possibility to simulate multi-scale hybrid models. The second major new feature is support of native BNGL, thus facilitating the comparison between non-spatial and spatial simulations of the same biochemical (BioNetGen) model.

I agree with the authors that the described new features represent a potentially major and relevant upgrade of MCell. At the same, I find that the manuscript is still quite unpolished and at least some parts of it appear to have been hastily written. Occasionally, the presentation is too thin and too vague to provide much guidance for a potential reader/MCell user.

Many of the provided tests are tests against either ODE or well-mixed stochastic models, such as SSA. In my view, that's fine, but I think it is important to emphasize in the manuscript that such tests, while providing an important prerequisite consistency check, do not represent a rigorous validation because they cannot say anything about the correct behavior in the fully stochastic, spatially inhomogeneous regime. Also, virtually all tests that are performed against another stochastic spatial simulator involve either MCell3 or MCell3-R. I think one should briefly explain, why these tests are not 'circular', although the underlying physics-simulation-engine does not seem to have changed from MCell3(-R) to MCell4.

I think that Section (Sec.) 3.3, as it stands, is not acceptable. The authors do not discuss the current state of the art at all and do not cite any references on hybrid simulations. Thus, they make it very hard for a potential reader to gauge what is actually shown in this Sec.

<u>Detailed comments:</u>

- page (p.) 3, line (l.) 29: "*…the currently maintained particle-based stochastic simulators that describes Smoldyn [7], eGFRD [8], SpringSaLaD [9], ReaDDy [10], and MCell3…*". Please point out that MCell3 is not based on a model of bimolecular reactions, such as the Smoluchowski model, in contrast to Smoldyn, eGFRD (Smoluchowski) and SpringSaLaD, ReaDDy (Doi).
- p.3, l.31: *"The typical simulation time-step in MCell is 1 µs,…"* Later the authors state "*…is given by a user-defined time step (usually 1 µs)."* (p.6, l.127). Does the MCell software assist the user in choosing the appropriate time step? Which are the criteria a user can draw on to determine if the model/simulation they consider is 'typical' or 'usual' and to choose the 'correct' time step.
- p.3, l.38: Please define 3DEM.
- p.3, l.44: "*MCell4 is a new C++ implementation of MCell,…".* Also*, "NFSim [14] is a C++ library…"* (p.4, l.66). Please specify the C++ standard that is meant here. Is it C++03 or one of the more recent ones, such as C++11, C++14 etc.?
- p.3, l.46: *"And most of MCell's features introduced previously [4] have been retained."* Please be more specific: Which features have not been retained? Why? Are these features deprecated or was it technically difficult to retain them? The authors might consider adding a table that provides an overview of the new features and removed features.

- p.4, l.68 "*then, a converter generates MDL, MDLR,…*" MDL is defined in Fig. 2's caption, but please define MDL, MDLR when they appear in the main text for the first time.
- p.4, l.76 "*This BNG library (libBNG) was designed…*" Is this a C++ library?
- p.4, l.78: "*libBNG does not support all special features and keywords of the BioNetGen tool suite yet,…*" Could a few of those features (other than BNGL functions) be explicitly mentioned as examples? Perhaps another table could be added that shows which BNG features are/are not supported by libBNG?
- p.4, l.80: "*And note that when needed, functions can be represented in MCell4's Python code.*" Does one need to add these functions every time one changes the corresponding BNGL file? Would that not reintroduce the issue that one was trying to overcome ("…*any potential changes made by hand to our MCell3-R model files will be lost.*" (p.4, l.71)*)*?
- *p.5, l.108: "Among the more advanced features introduced in MCell4 is the possibility to include transcellular and transmembrane interactions between surface molecules located on separate membranes."* As far as I can see, the authors neither provide an example nor discuss this topic any further. Despite of this, in the Summary (Sec. 4.1, p.25) the authors claim, "*As we have demonstrated here through example models, MCell4 has introduced many new features including…transmembrane or transcellular interactions between surface molecules.*" (p.25, l.402). I would suggest to either add a discussion of this topic and examples or to remove the statements related to transmembrane interactions.
- Sec. 2.2, p.7: The section headline promises 'a Closer Look', but I find the description somewhat too brief and too vague. In particular, please clarify if a user of MCell4 ever needs to use the API generator/YAML files? One could think so, because the authors state, "*To ensure…, the quality of the user experience when creating a model,… we have have* [sic] *developed a Python API generator,…*" (p.7, l.132). On the other hand, as far as I can see, the API generator is not discussed again and seems to play no role in the given examples, maybe suggesting that the API generator is 'only' relevant for developers. Please explain briefly, why the YAML format was chosen. Why was, as Fig. 4 indicates, pybind11 preferred over other solutions, such as Boost.Python or SWIG?
- Sec. 2.3: Is the described model structure (Fig. 5) enforced by the MCell4 software? If yes, please describe briefly how. If not, where can one find the coding style guide?
- Could the authors describe a little bit more the checkpointing features/capabilities of MCell4 mentioned in Fig.4?
- Fig.5: "*Model.py is the only required file.*" Required for what?
- Fig.7: The notation used for the Python search path suggests a Unix/Linux system. Does MCell4 also work with Windows? Also, perhaps, such more 'low-level' notes should be collected in a technical section (rather than a figure caption) placed at the end of the manuscript. Such a section could also provide information on the Python versions/packages required for MCell4. Speaking of Python, its installation can be tricky; is MCell4 available as a Docker image?
- p. 12, l. 215: Please define SSA and PLA and provide references.
- p. 13, l. 220: "*An MCell model is defined by a combination of Python and BNGL code.*" Is this statement correct for MCell4 exclusively? If so, pleased write 'MCell4' instead of 'MCell'.

Please double check that 'MCell4' is consistently used for all statements in the manuscript that apply to MCell4 only.

- p.13, l. 220: *"Although the recommended approach is to capture all the reaction rules and initial molecule releases using BNGL, it might be beneficial to use Python code for these definitions as well (e.g., to generate reaction networks programmatically)."* Here, I feel that a potential user is left alone with a too vague statement. Could the authors provide more specific guidance and expand on when it is preferrable to use Python code instead of BNGL? Similar remarks also apply to the statement "*There are also spatial model aspects that cannot be captured by BNGL."* Please provide examples. Also, the fact that a user must deal with two different languages (Python and BNGL) just to define a model, could that be considered as a potential drawback? Please discuss briefly this issue.
- p.13, l. 226: "*If no essential model aspects were skipped…"* Please provide examples of nonessential model aspects.
- Table 1, p.13: Please motivate the choice of unit for "MCell with BNG units/Volume-volume reaction rate". Why would one not just keep the MCell4 default unit for that case?
- Fig. 11, p.14: typo: 'sybsystem.bngl' → 'subsystem.bngl'
- p.15, l. 241: *"One can obtain identical results for MCell3/MCell3-R and MCell4 by using specific compilation options."* I find this confusing: Which compiler is meant here? A C++ compiler? The BioNetGen compiler? Also, why would the results depend on compiler-options? Please clarify and explain why those options are needed in more detail.
- Sect. 3.1.1, p.15: Some of the given numbers confuse me: "*The model is composed of 18 state variables, calcium ions and 63 reactions."* (l. 255). However, the caption of Fig. 13 (A) says: "*It consists of 36 states…*" Also, it is stated "*…five **s**, that represent the binding site for calcium molecules in the synchronous sensor; two **a** components that represent the binding sites for calcium in the asynchronous sensor…".* (l. 259). A compatible statement can be found in the caption of Fig. 13 (A) "*…which can be in five and two different states…"* However, the BNGL code (Fig. 12) and the actual Fig. 13 (A) show that **s** has 6 states (~0…~5) and **a** has 3 states (~0…~2). Please clarify.
- Sec. 3.1.2, p.17: Are the callback functions considered to be part of the model? If so, where do they fit in the set of files shown in Fig. 5? Is 'customization.py' (p.18, l.294) part of the model? It is not shown in Fig. 5, why not?
- Also, can a model/code that employs callback functions be exported into BNGL?
- P.18, l.301: typo $\frac{18^{12}}{12} \approx 10^{12}$ → $\frac{18^{12}}{12} \approx 10^{14}$
- P.18, l.314: typo: Figure 15 → Figure 15 C
- Please define PSD in Fig.15 A and C
- Sec. 3.1.3: Perhaps it would be worthwhile to illustrate the simulated geometry compartments in a simple figure?
- Sec. 3.1.4, 3.1.5: These sections are, in my view, somewhat too thin and appear to have been hastily written. I am aware that the considered models appear in Ref. [34], but I think one could nevertheless provide a little bit more of background/motivation to keep the manuscript more self-contained. Also, if I am not mistaken, Fig.16 is not referenced at all in the main text. Its caption mentions the NERDDS simulator, but no reference is provided. And nowhere in the manuscript is it stated what kind of simulations it provides. Stochastic or

deterministic, non-spatial or spatially resolved? Please also provide a reference for VCell when it appears first in the manuscript.

- Sec. 3.2: Please add the following pieces of information: The operating system, C++-compiler, optimization-level, python-version and benchmark-tool that have been used for the benchmarks (Fig. 18).
- Fig. 18: "*Both MCell3 and MCell4 use a single execution thread.*" Just to clarify: Does MCell4 support multi-threading? If so, which multi-threading library is used? If it does not, why?
- p.20, l.351: "*…for polymerization used in the SynGAP with TARP model.*" Please explain briefly.
- Sec. 3.3: Here, it seems to me that no attempt whatsoever has been made to discuss the current state of the art; no references have been provided. Thus, a potential reader will have a hard time to assess what has been achieved in this Sec., how the presented example relates to other approaches to hybrid simulations and what MCell4's current limitations are and what still needs to be done regarding hybrid simulations. In my view, it should be emphasized that the presented simple model does not represent a rigorous validation/theoretical justification. As an example of a more rigorous validation, see "Schaff JC, Gao F, Li Y, Novak IL, Slepchenko BM (2016) Numerical Approach to Spatial Deterministic-Stochastic Models Arising in Cell Biology. PLoS Comput Biol 12(12): e1005236" and references given therein. Please provide in this Sec. a more thorough discussion.
- Fig. 21: typo: the comment after the initialization of T_STEP: # in us → # in s
- Fig. 22: The authors observe that for slow diffusion the pure particle-based simulations show the fastest oscillations. Can this be understood from a theoretical point of view? Or is it counterintuitive? Could this behavior have any biological significance? Note that in the main text it is not even mentioned that one obtains deviations from SSA etc for the case of slow diffusion.
- p. 21, l.359: "*…using a differential equation…*" ODE or PDE?
- p.22, l.373: "*In the hybrid model, protein R is simulated as a concentration…*" Why was R chosen? Why not A?
- Fig. 19: The unit of the on-rate of the bimolecular reactions seems to be $M^{-1}s^{-1}$. Why not $\mu m^3 N^{-1} s^{-1}$, see Table 1 and Fig. 10?
- Fig. 20: The bimolecular reaction is now treated as a unimolecular one. Why? Is this a limitation of the current approach?
- p. 22, l. 384: "*…for the fastest reactions.*" Which reactions are the fastest?
- p. 22, l.383: "*Allowing 5x longer time step…*", p.24, l.385: "*Note that the time step for the particle-only model has to be 10−7s to precisely model these fast reactions.*" Perhaps one could use this example to explain how to decide on the time step size.
- Sec. 4.1, p.25: Given my previous comments, I think that several statements made in the Summary sound too absolute, such as "*This powerful new feature allows construction and execution of multi-scale hybrid models.*" (l.396), "*This allows a seamless transition between MCell4 and BNG simulation environments…*" (l.399), "*…the ability to go back and forth between MCell4 and BNG environments, and transmembrane or transcellular interactions between surface molecules.*" (l. 403). I would recommend qualifying those statements

accordingly and to point out/discuss current limitations as well. The same applies to similar statements made in the Abstract and in the Introduction.
