# Supplementary material for: MCell4 with BioNetGen: A Monte Carlo simulator of rule-based reaction-diffusion systems with Python interface
Source: PLoS Comput Biol. 2024 Apr 24;20(4):e1011800. doi: 10.1371/journal.pcbi.1011800 (PMC11073787; doi:10.1371/journal.pcbi.1011800)
Supplement: Attachment — Submitted filename: mcell4_article_response_to_reviewers_1.pdf [file pcbi.1011800.s003.pdf]

Dear Editors and Reviewers:

We would like to thank each of you for your thoughtful, thorough, and constructive comments on our manuscript. We have taken your helpful critiques into consideration and thoroughly revised our manuscript to address your suggestions. We believe your comments have contributed to a much stronger manuscript. Please find our revised version of the manuscript attached (with all revisions in blue text), and our replies to all of the Editor's and Reviewers' comments below (also in blue text).

Reviewer #1:

This paper describes a new version of MCell, version 4, which is a substantial overhaul of the prior versions, MCell3 and MCell3-R. MCell is a widely-used particle-based cell biology simulator. One major new feature is that the reaction entry method has been changed from MCell's model description language (MDL) to the BNGL language, which supports rule-based modeling and is widely supported. Another major new feature is a Python application programming interface. Both of these are quite valuable, making this a useful update to the software. The new features are demonstrated in this article through examples for SNARE complexes, CamKII modeling, and others. Although not the primary focus of this paper, it also describes a new library interface for the BioNetGen software, which is another important contribution.

Major issues

R1-1) Overall, this is important work that deserves to be published. However, it should be noted that nearly all of the capabilities described here have been supported by the Smoldyn software (which works at a similar level of detail) for several years, which is not mentioned in this paper.

R1-1a) The BNGL support was described in Andrews, 2017; that paper is cited here, although not in this context (Smoldyn's BNGL support is less integrated into the software than it is here).

R1-1b) Smoldyn's Python API was described in Singh and Andrews, Bioinformatics, 2021.

R1-1c) As with MCell4, Smoldyn's API also supports callback functions and multiscale simulations (e.g. a simulation was described in the 2021 paper that combined the Smoldyn and MOOSE simulators).

R1-1d) In addition, Smoldyn has supported transmembrane interactions since 2015 through the reaction_intersurface statement, which is described in the Smoldyn's User's Manual but has not been published elsewhere. The fact that these features are available in Smoldyn does not lessen their importance in MCell, but is something that readers should be made aware of.

We appreciate these comments and have added text in section 1.1 to inform the reader of these facts.

R1-2:Several BioNetGen extensions for spatial modeling were presumably developed in this work but are not described here.
In particular, how are diffusion coefficients, species names, and display parameters chosen for newly generated species?

A detailed description of how each of these is handled was given in our paper on Mcell3-R 2.1.3 (Tapia et al. 2019). I particular the calculation of diffusion constants for complexes is implemented in the MCell4 code here:

https://github.com/mcellteam/libbng/blob/1d9fe00a2cc9a8d223078aec2700e7b86b10426a/bng/species.cpp#L285

We have added a brief statement regarding these points in the text in section 2.4.3- Determination of Diffusion Constants for BNGL Complexes. Species names follow the same convention as in BNGL. There is experimental support for display of complex species in CellBlender.

See also our answer in R1-3.

R1-3: Are there situations where it's unclear whether newly generated species should be volume species or surface species and, if so, how is this handled?

There is never a case of ambiguity. If a complex contains any surface species, then the whole complex is considered to be a surface complex. Only the surface subunits are used to derive the surface diffusion constant. If there are no surface subunits, the complex is considered to be a volume complex. And all the volume subunits are included in the computation of the volume diffusion constant. The computation of diffusion constants of complexes is done according to equations published in Tapia et al., 2019.

We have added a brief description of diffusion constants and volume/surface species to section Determination of Diffusion Constants for BNGL Complexes.

R1-4: Also, does this version have backward compatibility with the comma and apostrophe notation that was used in MCell3-R?

CellBlender can export models as sets of files formatted as MCell3 MDL syntax and MCell3-R syntax (which uses the common and apostrophe notation) or formatted as a Python program for MCell4 (with reaction rules expressed in BNGL). The MCell4 software package includes a command-line executable that can run legacy MCell3 MDL and MCell3-R models and an importable Python module

to be used in models expressed as a Python program.

We have clarified this in the section 2.4.1 - “Extension of BNGL for Volume-Surface Reactions”.

R1-5:Does this BNGL implementation support generate-first, on-the-fly network expansion, or both?

Reaction rules are always evaluated using the “direct” method of NFsim as described in Sneddon et al., 2011. Perhaps this method corresponds to what you have called “on-the-fly network expansion”. In the direct method only an individual rule is resolved and evaluated, in just-in-time fashion, triggered by a pair of colliding species (a potential bimolecular reaction) or a single species (a potential unimolecular reaction). The result of the single rule expansion is stored in a flushable cache for time/memory efficiency. Thus it is critical to note that we never expand the whole network as this would fail in the case of pathological combinatorial explosion, for example with linear or branched polymers, or in case of complex molecules like CaMKII.

We have clarified this in the section 2.4 - ”Graph-Based Approach To Protein Modeling”.

R1-6:I didn’t understand section 2.2. I think it means that all of the Python API generation and the YAML code was simply a method for developing the Python API in the first place, and is not relevant for actual model development. Is this correct?

We have clarified that this is an important "Design and Implementation" detail relevant to developers. It unifies the C++ and Python APIs and the End User Documentation.
Added reference to the generated documentation and additional explanation to the caption of Fig 4.

R1-7: Also, how does the documentation fit into this development pipeline? Is this documentation for the end user, or only for software developers?

This is the documentation for the end user.
We have clarified by adding reference to the generated documentation in section 2.2.

R1-8: It might also be worth mentioning that this uses the pybind11 library, which isn’t mentioned here but appears in figure 4.

We have now mentioned this in the text in section 2.2.

R1-9:It took me a while to decipher Figure 8. In part, row G is listed between rows C and D. But also, is this figure necessary? I think it just describes how BioNetGen works, which wasn't part of the work described in this paper.

We have revised the figure's caption and explained the reason for including this figure in the paper. The figure describes how libBNG resolves and expands the individual reaction rule corresponding to a pair of colliding molecular species directly in just-in-time fashion. We have reorganized the figure to put row G at the end.

R1-10:I was pleased to see the extensive validation that was described in section 3.1. The text doesn't actually say that MCell4 always agreed with the other simulators, although this is implied. Did it always agree, or were there any notable exceptions?

This is stated at the beginning of the section "Testing and Validation" but for clarity we will also state: *"We did not encounter any statistically significant differences between MCell3, MCell3-R, MCell4 and BioNetGen (under the well-mixed assumption mentioned above)."*

R1-11: Also, is there some way for a reader to see these results in more detail, such as in the download package or in supplementary information? Are there additional concerns that a user should be aware of?

We have added a statement to the section "Testing & Validation":
All models used in this section for validation, including simulation output files, and scripts used to plot the data, are available in the article git repository [REF - https://github.com/mcellteam/article_mcell4_1].

R1-12:For the performance testing, it would be nice to see a test of the Michaelis-Menten benchmark model that was described in Andrews et al., PLoS Comp. Biol., 2010. This has become a relatively widely used benchmark, enabling comparison between different simulators. Admittedly, results depend strongly on the computer used, but they're still useful (or, better, is to compare results with other simulators using the same computer). Alternatively, the authors could run the models that they describe in Figure 18 on some other simulator, again with the goal of comparing performance across different simulators.

As suggested, we have run the MM benchmark with MCell3 and MCell4. In Andrews et al 2010, it is stated that MCell3 runs this benchmark in 120 s and Smoldyn runs it in 47 s. For further benchmarking here we used exactly the MCell3 model file published in Andrews et al., 2010. We ran the model on a 3.1GHz Intel Core i7 MacBook Pro (2017). As published, the model was set up to run with a time step of 1 ms for 10 s of simulated time (i.e. 10000 iterations). We found that MCell3 gives correct results and runs the model in 34 seconds. We are not sure of the reason for the much

longer benchmark time reported in Andrews et al. 2010. We then used CellBlender to build the identical model and exported it as an MCell4 (Python) model. Running in MCell4 gives correct results and runs the model in 28 s. We then further optimized the model settings in the MCell3 and MCell4 versions of the model. The maximum optimization we attempted used a time step of 1 s for 10 s of simulated time (i.e. just 10 iterations). Even at this very coarse time step we still obtained correct results. MCell3 ran the model in 0.4 s and MCell4 ran the model in 0.2 s.

Having run the benchmark, we are never-the-less torn on whether to include this benchmark in the main text or in the Supporting information. It is not clear what value this benchmark has in the present manuscript as it is a simple MM model under well-mixed conditions. As such it overlaps with several of the 350 unit tests that are already in the validation test suite for MCell4, tests which we have already stated are all passed by MCell4.

In general, we feel that it is difficult to run performance benchmarks in comparison with other simulators in a fair, rigorous, and authoritative way unless the authors of the other simulators collaborate in performing the tests. The goal of our manuscript is to introduce MCell4 to the community. Rigorous performance comparisons with other simulators should be the topic of a future collaborative article.

Minor issues

R1-13: The paper says that MCell4 is a new C++ implementation of MCell. Was it really rewritten from scratch? If so, this is a Herculean effort.

Indeed, MCell4 is an entirely new C++ implementation, written from scratch.

R1-14:I'm curious about the support for the traditional MDL language. Is it only retained for backward compatibility, or will it continue to be developed as new features are added? Also, to what extent will CellBlender be supported, since it's another easy-to-use option for non-experts.

MDL is still supported by the command-line executable included in the MCell4 package, but only for backward compatibility. We do not plan to update the features of MDL. MCell3 and MCell4 are both fully supported in CellBlender. We have added more details of support for MCell4 in CellBlender in section 1.5.

Grammar or spelling mistakes in lines: 122, 159, 184, 215, 241, 308, 353.

Corrected.

R1-15:Figure 1 is hard to see.

We have improved Figure 1. If this is still hard to see for you, please give specific recommendations.

Reviewed by Steve Andrews

Thank you Steve!

Reviewer #2:

The authors report a major new version of the stochastic simulation software MCell. MCell supports particle-based spatial models of biochemical systems. The new version, MCell4, features a Python API that opens the possibility to simulate multi-scale hybrid models. The second major new feature is support of native BNGL, thus facilitating the comparison between non-spatial and spatial simulations of the same biochemical (BioNetGen) model. I agree with the authors that the described new features represent a potentially major and relevant upgrade of MCell. At the same, I find that the manuscript is still quite unpolished and at least some parts of it appear to have been hastily written. Occasionally, the presentation is too thin and too vague to provide much guidance for a potential reader/MCell user.

R2-1: Many of the provided tests are tests against either ODE or well-mixed stochastic models, such as SSA. In my view, that's fine, but I think it is important to emphasize in the manuscript that such tests, while providing an important prerequisite consistency check, do not represent a rigorous validation because they cannot say anything about the correct behavior in the fully stochastic, spatially inhomogeneous regime. Also, virtually all tests that are performed against another stochastic spatial simulator involve either MCell3 or MCell3-R. I think one should briefly explain why these tests are not 'circular', although the underlying physics-simulation-engine does not seem to have changed from MCell3(-R) to MCell4.

It is true that some, but not all, of the validation tests and examples shown operate in the well-mixed regime. The goal in development of MCell4 was not to introduce new physics models, but rather to provide a more efficient variant of MCell3 with new features, and a means to couple to external physics engines. But please note that Figure 16 presents the validation of a spatially inhomogenous case comparing MCell4 to NERDSS, VCell PDEs, and MCell3, and that the hybrid model in section 3.3 points to the formation of spatial nanodomains when diffusion is slow.

A more complete set of spatially inhomogeneous/stochastic tests are part of the MCell4 test suite, included with the GitHub source code repository for MCell4. This test suite has been developed over decades of development and much of the previous validation testing of MCell3

has been reported in Kerr et al., 2008. The biophysics addressed in the test suite are independent of the simulation platform. Comparison of MCell4 to MCell3 is not circular because MCell3 and MCell-R are already validated using these same tests in the test suite. The tests themselves are the one single reference point of comparison for all versions of MCell. In particular, version 3 of MCell has been in continuous development, rigorously validated, and in active use by a large user community for fully two decades. Since MCell4 was rewritten from scratch, testing against the same test suite used for MCell3 and MCell3-R is really a type of cross-validation.

We have added a brief explanation of this in section 3.1.

R2-3: I think that Section (Sec.) 3.3, as it stands, is not acceptable. The authors do not discuss the current state of the art at all and do not cite any references on hybrid simulations. Thus, they make it very hard for a potential reader to gauge what is actually shown in this Sec.

We have added clarification and refs to improve this section. Our goal is simply to introduce this new feature of MCell4. The open and modular architecture we have implemented is compatible with the hybrid strategies already rigorously covered in the literature. We have now referenced these strategies in section 3.3. And in response to your important point R2-34 below, we have also added a discussion of the interesting results obtained with the hybrid model.

Detailed comments:

DONE: R2-4: page (p.) 3, line (l.) 29: "*…the currently maintained particle-based stochastic simulators that describes Smoldyn [7], eGFRD [8], SpringSaLaD [9], ReaDDy [10], and MCell3…*". Please point out that MCell3 is not based on a model of bimolecular reactions, such as the Smoluchowski model, in contrast to Smoldyn, eGFRD (Smoluchowski) and SpringSaLaD, ReaDDy (Doi).

MCell is indeed based on a model of bimolecular collisions and interactions and always has been. The model employs the Einstein-Smoluchowski rate of encounter and was first introduced in Bartol et al., 1991, and in more detail in Stiles and Bartol 2001, Stiles et al. 2001 (Synapses chapter) and Kerr et al 2008. Perhaps the reviewer's comment is based on a misunderstanding that seems to have appeared in the recent article by Johnson et al. 2021 MBOC.

We have added text to clarify this fact in section 1.1.

R2-5: p.3, l.31: "*The typical simulation time-step in MCell is 1 μs,…*" Later the authors state

“*…is given by a user-defined time step (usually 1 µs).*” (p.6, l.127). Does the MCell software assist the user in choosing the appropriate time step? Which are the criteria a user can draw on to determine if the model/simulation they consider is ‘typical’ or ‘usual’ and to choose the ‘correct’ time step.

At runtime MCell reports the reaction probabilities for bimolecular reactions and warns if probabilities are greater than 1. And at the end of the simulation MCell reports the estimated fraction of missed reactions caused by high probabilities.

We have added a brief statement explaining this in section 1.1.

R2-5: p.3, l.38: Please define 3DEM.

3DEM is 3-dimensional electron microscopy -- 3D reconstruction of cellular ultrastructure via serial-section electron microscopy. The abbreviation has been replaced in section 1.1.

R2-6: p.3, l.44: “*MCell4 is a new C++ implementation of MCell,…*”. Also, “*NFSim [14] is a C++ library…*” (p.4, l.66). Please specify the C++ standard that is meant here. Is it C++03 or one of the more recent ones, such as C++11, C++14 etc.?

MCell4 is implemented using the C++17 standard. Now stated in section 1.1.

R2-7: p.3, l.46: “*And most of MCell’s features introduced previously [4] have been retained.*” Please be more specific: Which features have not been retained? Why? Are these features deprecated or was it technically difficult to retain them? The authors might consider adding a table that provides an overview of the new features and removed features.

A table has been added to the Supporting Information - Section 5.

R2-8.1: p.4, l.68 “*then, a converter generates MDL, MDLR,…*” MDL is defined in Fig. 2’s caption, but please define MDL, MDLR when they appear in the main text for the first time.

Updated text in section 1.3

R2-8.2: p.4, l.76 “*This BNG library (libBNG) was designed…*” Is this a C++ library?

Updated text in section 1.3

R2-8.3: p.4, l.78: "*libBNG does not support all special features and keywords of the BioNetGen tool suite yet,…*" Could a few of those features (other than BNGL functions) be explicitly mentioned as examples? Perhaps another table could be added that shows which BNG features are/are not supported by libBNG?

A table has been added to the Supporting Information - Section 6.

R2-9: p.4, l.80: "*And note that when needed, functions can be represented in MCell4's Python code.*" Does one need to add these functions every time one changes the corresponding BNGL file? Would that not reintroduce the issue that one was trying to overcome ("…*any potential changes made by hand to our MCell3-R model files will be lost.*" (p.4, l.71))?

In MCell4, the BNGL is an integral part of the model and does not need to be converted into MDL-R format whenever the BNGL is modified (as is the case when using MCell3-R). Any customized Python functions that would need to act upon the BNGL for a model have access to all the content of the BNGL through the MCell4 Python API. MCell3-R has no API through which this would be possible. We have explained this better in the text in section 1.3.

R2-10: *p.5, l.108: "Among the more advanced features introduced in MCell4 is the possibility to include transcellular and transmembrane interactions between surface molecules located on separate membranes."* As far as I can see, the authors neither provide an example nor discuss this topic any further. Despite of this, in the Summary (Sec. 4.1, p.25) the authors claim, "*As we have demonstrated here through example models, MCell4 has introduced many new features including…transmembrane or transcellular interactions between surface molecules.*" (p.25, l.402). I would suggest to either add a discussion of this topic and examples or to remove the statements related to transmembrane interactions.

We thank the reviewer for making this important point. Due to space limitations we can't give detailed examples of every single new feature. We have added a statement to the beginning section 1.4 explaining that for those features not demonstrated in the results section, we have provided links to end-user documentation, tutorials, and source code repositories containing detailed information and validation tests. In particular we have revised the text regarding transmembrane interactions.

R2-11: Sec. 2.2, p.7: The section headline promises 'a Closer Look', but I find the description somewhat too brief and too vague. In particular, please clarify if a user of MCell4 ever needs

to use the API generator/YAML files? One could think so, because the authors state, "*To ensure…, the quality of the user experience when creating a model,… we have have* [sic] *developed a Python API generator,…"* (p.7, l.132). On the other hand, as far as I can see, the API generator is not discussed again and seems to play no role in the given examples, maybe suggesting that the API generator is 'only' relevant for developers. Please explain briefly, why the YAML format was chosen. Why was, as Fig. 4 indicates, pybind11 preferred over other solutions, such as Boost.Python or SWIG?

We have now made clear that the API generator is a "Design and Implementation" detail for developers only and improved the text in the caption in figure 4 to make it more relevant to users.

R2-12: Sec. 2.3: Is the described model structure (Fig. 5) enforced by the MCell4 software? If yes, please describe briefly how. If not, where can one find the coding style guide? • Could the authors describe a little bit more the checkpointing features/capabilities of MCell4 mentioned in Fig.4?

This model structure is not enforced by MCell4 itself. The MCell4 Python code generator in CellBlender follows this recommended structure. This has been clarified in the caption of Fig. 5.

R2-13: Fig.5: "*Model.py is the only required file."* Required for what?

This sentence has been removed.

R2-14: Fig.7: The notation used for the Python search path suggests a Unix/Linux system. Does MCell4 also work with Windows? Also, perhaps, such more 'low-level' notes should be collected in a technical section (rather than a figure caption) placed at the end of the manuscript. Such a section could also provide information on the Python versions/packages required for MCell4. Speaking of Python, its installation can be tricky; is MCell4 available as a Docker image?

We have Improved the caption of Fig 7.
Referencing the MCell4 installation documentation we have also added:
For ease of installation on all platforms, MCell4/CellBlender is provided as a "bundle" which includes Blender, CellBlender and MCell4 all packaged together. Blender comes with its own Python built in. MCell4 can use this Python.

R2-15: p. 12, l. 215: Please define SSA and PLA and provide references.

We have added references to section 2.4.2.

R2-16: p. 13, l. 220: "*An MCell model is defined by a combination of Python and BNGL code.*" Is this statement correct for MCell4 exclusively? If so, pleased write 'MCell4' instead of 'MCell'. Please double check that 'MCell4' is consistently used for all statements in the manuscript that apply to MCell4 only.

We have fixed this here and all references to MCell and MCell4 in the text.

R2-17: p.13, l. 220: *"Although the recommended approach is to capture all the reaction rules and initial molecule releases using BNGL, it might be beneficial to use Python code for these definitions as well (e.g., to generate reaction networks programmatically)."* Here, I feel that a potential user is left alone with a too vague statement. Could the authors provide more specific guidance and expand on when it is preferable to use Python code instead of BNGL? Similar remarks also apply to the statement "*There are also spatial model aspects that cannot be captured by BNGL.*" Please provide examples. Also, the fact that a user must deal with two different languages (Python and BNGL) just to define a model, could that be considered as a potential drawback? Please discuss briefly this issue.

We reworded this in the section 2.4.2 to say:
"The recommended approach is to define all the molecule species and reaction rules by BNGL, but the MCell4 Python API provides means to define them programmatically as well."

R2-18: p.13, l. 226: "*If no essential model aspects were skipped…*" Please provide examples of non essential model aspects.

This has been clarified in the section 2.4.2 with:
In general, no model component requiring specific location in 3D space can be exported to BNGL.

R2-19: Table 1, p.13: Please motivate the choice of unit for "MCell with BNG units/Volume-volume reaction rate". Why would one not just keep the MCell4 default unit for that case?

While BNG itself does not have a predefined unit systems, some of the solvers employed by BNG do require special attention regarding units. For example NFsim requires bimolecular

reaction rate constants in units of N^-1*s^-1, and the ODE and SSA solvers are most stable when supplied with units of um^3*N^-1*s^-1. We have clarified this in section 2.4.2.

R2-20: Fig. 11, p.14: typo: 'sybsystem.bngl' 'subsystem.bngl'

Fixed.

R2-21: p.15, l. 241: *"One can obtain identical results for MCell3/MCell3-R and MCell4 by using specific compilation options."* I find this confusing: Which compiler is meant here? A C++ compiler? The BioNetGen compiler? Also, why would the results depend on compiler options? Please clarify and explain why those options are needed in more detail.

This is accomplished by using specific macros that need to be enabled during compilation of MCell4 C++ source code. The options control how the random number stream is used at runtime. Certain optimizations in MCell4 result in different ordering of operations and different use of the random number stream. To get identical results, all operations that use the random number stream have to be performed in the same order. This has been clarified in the text in section 3.1

R2-22: Sect. 3.1.1, p.15: Some of the given numbers confuse me: "*The model is composed of 18 state variables, calcium ions and 63 reactions.*" (l. 255). However, the caption of Fig. 13 (A) says: "*It consists of 36 states…*" Also, it is stated "*…five **s**, that represent the binding site for calcium molecules in the synchronous sensor; two **a** components that represent the binding sites for calcium in the asynchronous sensor…*". (l. 259). A compatible statement can be found in the caption of Fig. 13 (A) "*…which can be in five and two different states…*" However, the BNGL code (Fig. 12) and the actual Fig. 13 (A) show that **s** has 6 states (~0…~5) and **a** has 3 states (~0…~2). Please clarify.

We thank the reviewer for catching these inconsistencies. There are actually a total of 36 states (6 for s, 3 for a, and 2 for dv), and 126 reactions. We have fixed and clarified these issues in the text of section 3.1.1.

R2-23: Sec. 3.1.2, p.17: Are the callback functions considered to be part of the model? If so, where do they fit in the set of files shown in Fig. 5? Is 'customization.py' (p.18, l.294) part of the model? It is not shown in Fig. 5, why not?

We have clarified this in the text and updated Fig. 5. There is an optional file called "customization.py" which we have added to Fig. 5. The purpose of this file is to override default behavior for CellBlender-generated code.

R2-24: Also, can a model/code that employs callback functions be exported into BNGL?

Added note that "An MCell4 model with callbacks cannot be exported to an equivalent BNGL representation."

R2-25: P.18, l.301: typo 18^12/12 ~= 10^4

Fixed.

R2-26: P.18, l.314: typo: Figure 15 □ Figure 15 C

Fixed.

R2-27: Please define PSD in Fig.15 A and C

PSD is "Post Synaptic Density". Now defined in the text.

R2-28: Sec. 3.1.3: Perhaps it would be worthwhile to illustrate the simulated geometry compartments in a simple figure?

We have added the requested illustration as Fig 15D.

R2-29: Sec. 3.1.4, 3.1.5: These sections are, in my view, somewhat too thin and appear to have been hastily written. I am aware that the considered models appear in Ref. [34], but I think one could nevertheless provide a liLle bit more of background/motivation to keep the manuscript more self-contained. Also, if I am not mistaken, Fig.16 is not referenced at all in the main text. Its caption mentions the NERDDS simulator, but no reference is provided. And nowhere in the manuscript is it stated what kind of simulations it provides. Stochastic or deterministic, non-spatial or spatially resolved? Please also provide a reference for VCell when it appears first in the manuscript.

We have now made reference to Fig. 16 This model shows that membrane localization can affect the speed and stability of protein complex formation and that this behavior cannot be modeled precisely with ODE methods. We have added refs and text about the NERDSS simulator and VCell.

We have added an explanation to section 3.1.5.

R2-30: Sec. 3.2: Please add the following pieces of information: The operating system, C++-compiler, optimization-level, python-version and benchmark-tool that have been used for the benchmarks (Fig. 18).

We have now stated in the text that execution times were measured on AMD Ryzen 9 3900X@3.8GHz, operating system Debian 10, compiler gcc 9.3, optimization level -O3, Python 3.8.

R2-31: Fig. 18: "*Both MCell3 and MCell4 use a single execution thread.*" Just to clarify: Does MCell4 support multi-threading? If so, which multi-threading library is used? If it does not, why? •

Both MCell3 and MCell4 use a single execution thread. The reason why no parallelization in MCell is needed is that usually the simulation is run in parallel with different random seeds and then individual trajectories and ensemble of trajectories are analyzed. Over the years we have collaborated with Computer Scientists (Scott Baden) and have attempted parallelization of MCell. The performance always suffered from Amdahl's Law where parts of sequential computations did not allow substantial speedup.

R2-32: p.20, l.351: "…*for polymerization used in the SynGAP with TARP model.*" Please explain briefly.

We revised this section and added a brief description and reference for this model in section 3.2.

R2-33: Sec. 3.3: Here, it seems to me that no attempt whatsoever has been made to discuss the current state of the art; no references have been provided. Thus, a potential reader will have a hard time to assess what has been achieved in this Sec., how the presented example relates to other approaches to hybrid simulations and what MCell4's current limitations are and what still needs to be done regarding hybrid simulations. In my view, it should be emphasized that the presented simple model does not represent a rigorous validation/theoretical justification. As an example of a more rigorous validation, see "Schaff JC, Gao F, Li Y, Novak IL, Slepchenko BM (2016) Numerical Approach to Spatial Deterministic Stochastic Models Arising in Cell Biology. PLoS Comput Biol 12(12): e1005236" and references given therein. Please provide in this Sec. a more thorough discussion.

See also our response to R2-3 above. The Python API of MCell4 is compatible with hybrid simulation methods described elsewhere. But it is beyond the scope of the current paper to describe these in great detail. In accordance with the reviewer's constructive suggestions we have revised our discussion of the current state of the art of hybrid model, referencing the literature.

R2-34: Fig. 21: typo: the comment a6er the initialization of T_STEP: # in us # in s • Fig. 22: The authors observe that for slow diffusion the pure particle-based simulations show the fastest oscillations. Can this be understood from a theoretical point of view? Or is it counterintuitive? Could this behavior have any biological significance? Note that in the main text it is not even mentioned that one obtains deviations from SSA etc for the case of slow diffusion.

We thank the reviewer for this very constructive question! Your question has pointed us to a very interesting result that we did not see at first. We have improved our discussion of this phenomenon in section 3.3.

R2-35: p. 21, l.359: "*…using a differential equation…*" ODE or PDE?

We are referring to ODE's. Now clarified in the text.

R2-36: p.22, l.373: "*In the hybrid model, protein R is simulated as a concentration…*" Why was R chosen? Why not A?

We have now explained this in the text of section 3.3.

R2-37: Fig. 19: The unit of the on-rate of the bimolecular reactions seems to be. Why not!, see Table 1 and Fig. 10?

Table 1 makes it clear that the default unit for bimolecular reactions is $M^{-1}s^{-1}$ and that the use of BNG units in MCell4 is optional.

R2-38: Fig. 20: The bimolecular reaction is now treated as a unimolecular one. Why? Is this a limitation of the current approach?

The reasoning here is now explained the text in section 3.3. Note that the bimolecular reaction labeled A_and_R_to_AR in the particle only model has been transformed into the pseudo

first-order reaction labeled A_to_R in the hybrid model where the pseudo first-order rate A_koff is updated during step 3 of the the simulation loop in Fig 21.

R2-39: p. 22, l. 384: "…*for the fastest reactions.*" Which reactions are the fastest? • p. 22, l.383: "*Allowing 5x longer 0me step…*", p.24, l.385: "*Note that the 0me step for the particle-only model has to be 10−7s to precisely model these fast reactions.*" Perhaps one could use this example to explain how to decide on the time step size.

We have revised our explanation of this in section 1.1 and 3.3.

R2-40: Sec. 4.1, p.25: Given my previous comments, I think that several statements made in the Summary sound too absolute, such as "*This powerful new feature allows construction and execution of multi-scale hybrid models.*" (l.396), "*This allows a seamless transi0on between MCell4 and BNG simula0on environments…*" (l.399), "*…the ability to go back and forth between MCell4 and BNG environments, and transmembrane or transcellular interactions between surface molecules.*" (l. 403). I would recommend qualifying those statements accordingly and to point out/discuss current limitations as well. The same applies to similar statements made in the Abstract and in the Introduction.

We have revised these statements according to your suggestions.

Reviewer #3:

In their article "MCell4 with BioNetGen: A Monte Carlo Simulator of Rule-Based Reaction-Diffusion-Systems with Python Interface" the authors Husar et al. deliver a detailed description of the new technical features of the MCell4 simulation environment and exemplify its capibilities by simulating several biochemical models, which at once validate the MCell4 simulations with respect to previous simulation frameworks and ODE models and are used for a comparison of the computational performance of the new framework.

Without any doubt, the authors present a highly useful extension that largely enhances the capabilities of an important biochemical simulation framework, which renders their work highly relevant to the computational biology community and beyond. In particular the combination of MCell with BioNetGen is a major step forward and highly augments the versatility of this simulation tool. The article therefore fits very well into the spectrum of PLoS Computational Biology and would constitute an excellent and interesting contribution once adequately adapted.

R3-1: I found it a bit harder to come to clearly positive conclusion on the form of presentation, i.e. the way the content is composed and presented. In my opinion, in its current form, the focus

of the article is still too technical. The technical extensions are presented in great detail, sometimes up to the point that the names of concrete Python routines are mentioned in the main text.

Our article is submitted as a PLoSCompBio “Software” article which is intended to cover the technical aspects of the “Design and Implementation” of MCell4 with BioNetGen, as well as presentation of examples of how to use the software. Some of these details are intended for developers of simulators and software tools in the field of Computational Biology. The title of the “Methods” section should have been set to “Design and Implementation”. This has been corrected in the revised document.

R3-2: On the other hand, while the authors present a decent range of simulated example systems, the description of these models and in particular of the simulation results is a bit short, sometimes almost being reduced to one sentence mentioning good agreement between MCell4 sims and previous approaches. This part should be expanded and rewritten with a stronger focus on the biological aspects, while details such as the part on specific callback functions used should be outsourced into a supporting text.

We have revised the text in accordance with these suggestions.

R3-3: Also the organization of the figures should be changed. 23 main figures, of which most are code fragments, seem inappropriate for the format of a PLoS Comp Biol article. Many figures could be grouped together into separate panels of one figure (such as, e.g., Figs. 2 and 3 and Figs. 4--6; btw., please enlarge the arrow heads in these figures). The authors should consider, again, outsourcing some of the figures into a supporting document; some of the figures showing code fragments may be good candidates for this. There is also need for improving some of the captions. For example, the caption of Fig. 18 does not clearly refer to its separate panels A and B.

Again, our manuscript is a PLoS Software article. We have revised and reorganized the text. We have enlarged the arrows in Figs. 2-6. We have Improved caption of Fig. 18

R3-4: The discussion / conclusion section is the weakest part of the article. It is too short, does not talk about the performance and accuracy of the new simulator, and also not about the biological systems that were simulated. It mainly focuses on future directions / extensions. Also there is no comparison between MCell4 and other prominent simulation schemes. What are the advantages / disadvantages of using MCell4 / preferring it above other schemes? This part should be thoroughly rewritten and expanded.

We have revised the Conclusion section.

R3-5: In terms of language, the article is generally well written, but still contains many occurrences of stylistic and linguistic shortcomings, such as missing articles or other words, or usage of singular where plural should be used, up to the point that I found it hard to believe that the mother tongue speakers in the author list read the article with the intention of fixing such issues. I will not list these minor issues here because I believe that large parts of the article need to be rewritten with a different focus anyhow. But in a new version of the article, all authors should make an effort in ensuring that the article is well written and uses correct wording. Issues such as missing articles or swapped words should not be fixed by reviewers or copy editors.

This has now been fixed throughout the entire text.

R3-6: I therefore recommend a thorough revision of the article with a particular emphasis on reducing / outsourcing some of the technical details, while rewriting the part on example simulations with a stronger focus on the biological aspects and results (even when these agree with previous results); this would make the article also attractive and more accessible to biologists and experimentalists, while in its current form it mainly targets experts of biochemical simulator development, i.e. mainly computer scientists.At the end I am listing some specific issues and questions that should be considered and fixed in a new version of the article:

Again our paper is a “Software” article. But we have tried to address these comments as much as possible in the text.

R3-7: - p.2: "... in the spatial and temporal scales of nm to 10s of uM and us to 10s of seconds": Sorry, but it is impossible to understand what is meant here. Please rewrite this.

Fixed.

R3-8: - p.3: "as an individual agent": this can be confusing, since many readers will think of agent-based simulations here. Perhaps it would be better to talk of invdividual particles / spheres.

We have now stated that particles are spatially resolved agents. It should be understood that MCell is an agent-based simulator, where the agents are spatially resolved, aka "particles". Particle-based simulations are a subset of agent-based simulations.

R3-9: - p.3: The text suggests that there is no volume exclusion for volume particles, but there is

one on the membranes. Is this indeed so? If yes, why?

We have now explained that this is a limitation of the current implementation in section 4.2. But has large performance advantages for systems that are not strongly crowded and/or for small diffusing particles even in crowded systems.

R3-10: - p.3: "it is significantly faster": significantly faster can mean factor 1.2x faster, but it seems that the performance enhancement can be far higher in MCell4. I would therefore replace "significantly" by "dramatically" or similar, and name a concrete number / speed-up factor here.

Thanks, we agree. We have now stated it this way. (But please note your previous statement that we did not discuss performance).

R3-11: - p.3: "And most of MCell's features ... have been retained.": Why "only" most, and not all? Was there technical problems, or were they deemed irrelevant?- p.5/6: "molecule-wall collision": I would rather say "molecule-surface collision". Earlier "membranes" is used synonymously with "surfaces". It would be good to settle for one term here ("surfaces" seems most appropriate).

We have revised the text according to these suggestions, we also added a new supplementary section with differences in features between MCell3 and MCell4.

R3-12: - p.6: What exactly is meant by "multi-physics" simulations? I only know this term from game software development, as in natural sciences simulations there should be only one physics (that arguably can be implemented in different ways, with varying degrees of accuracy).

This is a widely accepted term in the field of numerical simulation (see https://en.wikipedia.org/wiki/Multiphysics_simulation). In fact, there is a new journal called "Multi-Physics".

R3-13-1: - p.7: Why is the "DiffuseReactEvent" second in the schematic, but fourth in the caption? This is confusing. Please, on occasion, increase the arrow heads in all of these schematics.

Fixed

R3-13-2- p.9: 3^10 seems an impressively high number, but it is actually "only" ~60.000... It would be good to write this out in order to avoid unjustified superlatives here.

It's exactly 59049. Fixed.

R3-13-3- Fig. 6: What does "not all dependencies are shown" mean here? This seems strange...

This is with respect to Fig. 5 that shows all file dependencies.

R3-14: - p. 12: The notation with comma/apostrophe for inwards/outwards is introduced twice and therefore repeating. Please check.

This has been fixed in section 2.4.1

R3-15: - p. 15: tilde notation for states ~0 and ~1: Why the choice of the tilde, and not simply state "0" and "1"?

The tilde is BNGL notation. Now noted in the text.

R3-16: - p. 17: Description of callback functions and reference links to code: This should not be part of the Results section, because it describes _Methods_. The results section here should be talking of the SNARE complex system and what we learn about it by using MCell4 simulations. Certainly, the results section of a PLoS Comp Biol article should not talk of particular Python classes that were implemented for obtaining some results. This purely technical content should part of a Methods section or the SI.

Callbacks are first mentioned in section 1.4.4. Inserting an expanded description of callbacks into the Design and Implementation section would lead to an orphaned subsection. We believe that the current location makes the article easier to follow. This example is intended to demonstrate the specifics of how to use Callbacks to implement release of Glutamate, driven by the dynamics of the SNARE complex, which we feel is of general interest users of MCell4.

R3-17: - p. 19: 17000 molecules / um^2: This appears a rather high molecule number. Is this the standard density usually simulated in MCell4? Then this should be mentioned. It remains a bit unclear, why for such a high density the surface is tiled into 40000 tiles / um^2 if only one molecule can occupy a tile at a time. Is it, in this setting, not highly probable that two molecules

could end up in one tile?

The density comes from the benchmark model we are using here. We have now explained that these settings allow free diffusion of surface molecules, as required for this model.

R3-18: - p. 21: "A demonstration that will be shown in this section ...": "Showing" and "demonstrating" are highly synonymous, so it seems that this introduction can be shortened. E.g. by saying "Here we show" or "Here we demonstrate this via ..."

Fixed.

R3-19: - p. 22, Figs. 19 and 20: This can clearly be grouped into one figure. Please indicate what "protein R" is in the captions.

We have improved the captions of figures 19 and 20.

R3-20: - p. 22: Why is cm^2/s used as unit here, and not the "canonical" MCell4 units grouped in Table 1?

Table 1 shows mainly the reaction rate units, the diffusion constant unit, cm^2/s is listed in the caption of Table 1.

R3-21: - p. 22/23: The sentence starting with "This is thanks to: " should be rewritten, as it does not read well right now. The side comments should be removed from the main listing of "reasons".

Fixed.

R3-22: -p. 25: The last paragraph of section 4.1 is way to short and vague. The improvements in simulation speed, system size etc. should be explicitly mentioned with (possibly order of magnitude) numbers here.

We have added a statement that the simulation speed is about 10x faster for models with large reaction networks.

R3-23: - p. 25: "MCell4 does not support the definition of spatially extended complexes ...": This

is very confusing, as it can understood as impossibility of including any spatial structures in MCell4. Please rewrite this more to the point.

We have clarified that MCell4 does not currently model the spatial shapes of assembled BNGL complexes.

—---

Figure Files:
While revising your submission, please upload your figure files to the Preflight Analysis and Conversion Engine (PACE) digital diagnostic tool, https://pacev2.apexcovantage.com. PACE helps ensure that figures meet PLOS requirements. To use PACE, you must first register as a user. Then, login and navigate to the UPLOAD tab, where you will find detailed instructions on how to use the tool. If you encounter any issues or have any questions when using PACE, please email us at figures@plos.org.
