# Supplementary material for: MCell4 with BioNetGen: A Monte Carlo simulator of rule-based reaction-diffusion systems with Python interface
Source: PLoS Comput Biol. 2024 Apr 24;20(4):e1011800. doi: 10.1371/journal.pcbi.1011800 (PMC11073787; doi:10.1371/journal.pcbi.1011800)
Supplement: Attachment — Submitted filename: review_final.pdf [file pcbi.1011800.s004.pdf]

I think that overall, all raised issues have been addressed satisfactorily and I recommend publication.

A few remaining minor issues:

1. Please provide the benchmark-tool you used, see R2-30.
2. I find the new statement *"Together, these improvements allow simulation of new classes of systems that could not be modeled previously, <u>especially systems that exhibit stochastic fluctuations, spatial nanodomains of particles, and combinatorial complexity</u>."* (p. 29) somewhat misleading, also in light of Steve Andrews' comment R1-1. Please list here features that are new in MCell4 (not the modeling of stochastic fluctuations).
3. Please define "Einstein-Smoluchowski rate of encounter" (p. 3). Note that this term was not used in Kerr et al 2008 as far as I can see, and it is somewhat ambiguous.

Finally, I would like to comment on the authors' response to R2-4 (just to clarify—no further action required). I am aware of the Johnson et al 2021 MBOC article, but similar statements have been made elsewhere. In Johnson and Hummer 2014 Phys Rev X one can find the following characterization:
"Several methods designed to reach a full cellular scale by taking larger time steps (microsecond to millisecond) replace reactive collisions with phenomenological probability models that are derived to reproduce, for example, the bulk reaction rate [20, (Kerr et al 2008)]. The dynamics of these rule-based numerical approaches, however, is not exactly governed by any PDEs and therefore the behavior of the system may not faithfully reproduce the behavior of BD simulations or results from concentration-based methods."
